# Exposure of cane toad hatchlings to older conspecifics suppresses chemosensory food tracking behaviour and increases risk of predation post-exposure

**DOI:** 10.1371/journal.pone.0233653

**Published:** 2020-05-29

**Authors:** Samantha McCann, Michael Crossland, Richard Shine

**Affiliations:** School of Life and Environmental Sciences, University of Sydney, Sydney, NSW, Australia; Universitat Bremen, GERMANY

## Abstract

Attempts to control invasive species using species-specific pheromones need to incorporate an understanding of interactive effects among those pathways. The larvae of invasive cane toads (*Rhinella marina*) utilise chemical cues to repulse, attract or suppress conspecific larvae. We can exploit these effects to reduce toad abundance, but the effects of each cue may not be additive. That is, exposure to one type of cue may lessen the impact of exposure to another cue. To assess this possibility, we exposed toad larvae to combinations of cues. Tadpoles that had been exposed to the suppression cue during larval development exhibited no response to the attraction cue, resulting in lower capture rates in attractant-baited traps. Suppression, however, did not affect a tadpole’s response to the alarm cue, and exposure to the alarm cue during tadpole development did not affect response to the attraction cue. Tadpoles exposed to the suppression cue were smaller than control tadpoles at 10 days post-exposure, and consequently were more vulnerable to gape-limited invertebrate predators. Our results demonstrate that the responses by toad tadpoles to chemical cues interact in important ways, and are not simply additive when combined. Control efforts need to incorporate an understanding of such interactions if we are to most effectively use chemical-communication pathways to control invasive amphibians.

## Introduction

Increasing rates of international commerce and exchange are translocating more and more species into areas far outside their native ranges [1]. Although most of the species that are transported fail to establish in their new homes, invasions nonetheless can impose substantial ecological, evolutionary and economic impacts [1–4]. As a result, we urgently need effective ways to eradicate or manage invasive populations [5] without deleteriously affecting native species that occupy the same habitat [6]. To achieve species-specificity and thus avoid collateral damage, we may be able to exploit species-specific communication pathways and competitive mechanisms [7–10].

Many aquatic species–including invasive taxa–utilise chemical communication systems, and chemicals that function in this way provide potential avenues with which to target aquatic invaders without affecting native by-standers [11]. A wide range of chemical cues is used to locate food and mates, avoid predators, and engage in social interactions [6]. We may be able to manipulate such signals in order to evoke specific behavioural or chemical responses from a target species. For example, traps baited with conspecific sex pheromones are being used to capture invasive sea lampreys (*Petromyzon marinus)* in North America [12] and invasive signal crayfish (*Pacifastacus leniusculus*) in Britain [11]. By using species-specific sex pheromones as the “lure”, by-catch of native species is reduced. Chemical-based attractants can just as easily be applied to species that spend only part of their life cycle in aquatic environments, even if the aquatic phase is non-reproductive (e.g. many amphibians [9]).

Cane toads (*Rhinella marina*) are one of the world’s most intensively studied invasive taxa. The species is native to large areas of Central and South America, but has been introduced to more than 40 countries in attempts at pest control [13]. In Australia, cane toads have had a devastating impact on native fauna, by fatally poisoning frog-eating predators [14]. Most efforts at toad control have targeted toads in their terrestrial adult stage (e.g. manual removal [15]; traps [16]) but recent research has identified a new approach–manipulating the chemical communication systems used by toad tadpoles [17–19]. This approach exploits two important aspects of toad biology: reliance upon water bodies for breeding, and the extensive phylogenetic divergence between cane toads and native Australian anurans (a divergence that has reduced any impact of the toads’ chemical communication systems on the larvae of native anurans [20, 21]).

At least three types of chemical cues used by cane toad tadpoles may have potential for invader control. If encountered by toad hatchlings, waterborne cues produced by older larval conspecifics (the ‘suppression cue’ [22]) can dramatically reduce subsequent rates of growth, survival and development of the tadpoles that develop from those hatchlings [18, 21]. Cane toad tadpoles also exhibit avoidance responses to chemical cues from crushed conspecifics (the ‘alarm cue’ [23]); tadpoles continuously exposed to the alarm cue exhibit reduced rates of survival and smaller size at metamorphosis [24]. Finally, the ‘attraction cue’ (bufadienolide toxins present in cane toad eggs) attracts tadpoles to a conspecific egg clutch, eliciting a rapid approach and intense cannibalism [22]. We can exploit that response by using toxin squeezed from adult toad parotoid glands (containing bufadienolide toxins present in toad eggs) as the bait in funnel traps to catch and remove toad tadpoles from natural water bodies [25].

All three of these chemical cues thus have the potential to play some role in controlling invasive cane toads in the larval phase–by suppressing their development, reducing their survival, or luring them into traps. The current paper addresses a critical question that must be answered before we can work out how to utilise these chemically-mediated responses for toad control: how do responses to these cues interact? Plausibly, for example, toad tadpoles exposed to the alarm cue might focus on escaping the perceived threat of predation rather than searching out and consuming newly-laid toad eggs (and hence, become less responsive to the attractant cue); or tadpoles whose developmental trajectories have been modified by exposure to the suppression cue might thereafter fail to respond to alarm or attraction cues. We thus conducted experiments to explore the ways in which exposure to one type of chemical cue influences larval responses to other kinds of chemical stimuli, in order to inform the development of maximally effective methods for toad control.

## Materials and methods

This study was approved by the University of Sydney Animal Care and Ethics Committee under Protocol Number 2013/6033.

### Toad breeding

Adult cane toads were collected by hand from sites in Western Australia (WA: Kununurra– 15.778477, 128.744960; Doongan– 15.390413, 126.293106) and the Northern Territory (NT: Middle Point– 12.579564, 131.313918; Mary River Park– 12.904422, 131.651241) and brought back to our laboratory at Middle Point. Pairs of adult toads from each locality were injected with leuprorelin acetate (Lucrin, Abbott Australasia) to induce breeding (see Hayes *et al*. [26] for detailed methods). Newly laid clutches of eggs were kept in individual 18 L tubs in unchlorinated bore water at room temperature (25°C) and aerated until they reached the stages required for our experimental trials.

### Effects of exposure to the suppression cue

#### Suppression treatment

Plastic aquaria (1 L, 120mm x 170mm x 68mm) each holding 10 hatchlings (Gosner stage 18, not yet free swimming, Gosner [27]) in 750 mL of bore water, were assigned to either a ‘suppression’ or ‘control’ treatment. We placed a flyscreen mesh container (50mm x 70mm x 20mm, holes 1 mm x 1 mm) into the water in each aquarium, and added three live cane toad tadpoles (Gosner stage 29–33, previously acquired from an older Middle Point clutch) to the container for each of the ‘suppression’ aquaria. In ‘control’ aquaria the mesh container remained empty. The mesh containers were left in place for approximately 48 h, until the hatchlings reached Gosner stage 25 (i.e. had developed into free-swimming tadpoles). Five of those free-swimming tadpoles were randomly selected from each aquarium, and transferred into new aquaria with fresh bore water. They were fed with crushed algae wafers each day (to excess), and we changed the water every second day. These tadpoles were the source of ‘suppression’ and ‘control’ treatment tadpoles for the experiments detailed below. Each tadpole was used in only one of the following three types of trials.

#### Trapping rates

Ten days post-exposure, 20 tadpoles from each treatment (from a mixture of holding aquaria) were randomly allocated to trays of water (680 x 420 x 70 mm), and left to acclimate for one hour. These trays were assigned to either ‘toxin trapping’ or ‘no toxin trapping’ (control), creating the following four treatments: ‘suppressed + toxin trap’, ‘suppressed + control’, ‘not suppressed + toxin trap’ and ‘not suppressed + control’ (for 3 clutches, with 3–5 replicates each). Tadpole traps were transparent 1 L plastic containers (120mm x 170mm x 68mm) with one wall containing an inward-pointing funnel (funnel: length 35 mm, largest diameter 44 mm, smallest diameter 12 mm), with one trap per tray. Traps were placed against the middle of the smaller wall of the tray, with the opening of the funnel facing into the tray. Toxin baits (attractant cues) were made by squeezing 200 mg of fresh toxin from the parotoid glands of adult female toads into a cup filled with 30 mL of bore water (on the advice of Crossland et al. [17]). The bait was left to sit for half an hour at room temperature before being added to the trap in the ‘toxin trapping’ treatments; an equivalent volume of bore water was simultaneously added to the control traps. The number of tadpoles inside each trap was counted after 90 min.

We also repeated these trials as a separate experiment (using different tadpoles, suppressed and not suppressed), using food (0.5 g of algae wafers) as a bait to determine whether patterns observed in the above trials were seen in response to cues from other types of food as well as in response to the conspecific attractant cue. It should be noted that whilst directly comparing the attractiveness of equal concentrations of food bait and toxin bait to tadpoles would have been informative, the difficulty in accurately doing so was the reason the cues were not compared within a single experiment. Firstly, the concentration of bufanolide toxins within parotoid exudate can be variable and difficult to measure, requiring chemical tests that would render the toxin no longer useful to the experiment. Secondly, the strength of the response of tadpoles (i.e. the number of tadpoles captured) to the conspecific attractant cue can vary widely, due to abiotic conditions, or often without obvious reason. While the factors influencing the strength of this response are currently being studied, this variation in response makes it difficult to determine the ‘strength’ of an attractant cue. For these reasons the attractiveness of tadpoles to toxin bait and food bait are not directly compared.

#### Response to alarm cues

Twelve days post-exposure, 20 tadpoles from each suppression treatment were placed into individual 1 L plastic aquaria (120mm x 170mm x 68mm) filled with 250 mL of bore water, and left to acclimate for 1 h. Aquaria were assigned to either ‘alarm cue’ or ‘water cue’ (control), such that there was a total of four treatments; ‘suppressed + alarm cue’, ‘suppressed + control’, ‘not suppressed + alarm cue’ and ‘not suppressed + control’ (3 clutches, 10 replicates of each treatment). The alarm cue was prepared by rapidly macerating 3 g of live conspecific tadpoles, combined with 20 mL of bore water, and filtering the mixture through a nylon mesh net (holes 200 μm). A drop of food colouring was added to enable us to see the alarm cue dispersing through water. The control cue consisted of 20 mL of bore water with a drop of food colouring. A syringe was used to inject 1 mL of either the alarm or control cue into the water along the edge of each aquaria. The tadpole in the aquaria was encouraged to swim towards the cue by gently touching its tail. The tadpole’s reaction as it encountered the cue was scored as either ‘repulsed’ (rapid u-turn, sudden onset of immobility and dropping to the bottom of the aquaria, or suddenly swimming rapidly around the perimeter of the aquaria) or ‘not repulsed’ (tadpoles continued to behave as they had been before encountering the cue). Each tadpole was encouraged to encounter the cue three times, and the proportion of times it was ‘repulsed’ was recorded.

#### Vulnerability to predation

Two days post-exposure, 10 tadpoles from each suppression treatment were weighed (g) and randomly allocated to plastic tubs (360 x 180 x 200 mm) filled to a depth of 10 cm with bore water (4 clutches, 6–8 replicates per clutch per treatment). Tadpoles were left to acclimate for 1 h. At 0700 h, we added one water bug (*Diplonychus* sp.) to each tub, along with a floating stick (100 mm long) to enable the bug to rest at the top of the water. The number of tadpoles consumed by the bug was monitored for 24 h. At 10 days post-exposure, these trials were repeated with other tadpoles (same clutches, 4–8 replicates per clutch per treatment).

### Effects of exposure to the alarm cue

#### Alarm treatment

Cane toad hatchlings (3 clutches, Gosner stage 18–19) were placed into 1 L clear plastic aquaria (10 per tub) holding 750 mL of bore water, and left to reach Gosner stage 25. Five viable tadpoles from each aquarium were then randomly selected and placed into a new aquarium. Half of these aquaria were allocated to the ‘alarm’ treatment, and half to the ‘control’ treatment. Alarm cue was prepared as above, but without the addition of dye. Each morning for 7 days, we added 1 mL of alarm cue into each of the ‘alarm’ aquaria, and 1 mL of bore water into the ‘control’ aquaria. We fed the tadpoles crushed algae wafers each afternoon, and changed the water every second day.

#### Trapping rates

Eight days post-exposure, 20 tadpoles from each treatment were randomly assigned to trays of bore water, and left to acclimate for 1 h. These trays were assigned to either ‘toxin trapping’ or ‘no toxin trapping’ (control), such that there was a total of four treatments: ‘alarm + toxin trap’, ‘alarm + control’, ‘control + toxin trap’ and ‘control + control’ (2–5 replicates each). Toxin baits were made and trapping trials carried out as described above.

### Statistical analysis

To analyse the data on trapping rates, we used logistic regression (see Warton & Hui [28]) in RStudio [29], with the number of tadpoles trapped (versus not trapped) as the dependent variable and with ‘suppression treatment’ and ‘trapping treatment’ as fixed effects. The analysis was based on the quasi-binomial distribution to account for over-dispersion of data. We also used logistic regression to compare the number of tadpoles trapped versus not trapped using a food bait (algae wafer), based on the binomial distribution. Tadpole clutch identity was included in both analyses as a random factor.

To analyse the data on repulsion, we used logistic regression to compare the number of tadpoles repulsed versus not repulsed, using ‘suppression treatment’ and ‘alarm treatment’ as fixed effects. The analysis was based on the quasi-binomial distribution to account for over-dispersion of data. Tadpole clutch identity was included as a random factor.

To analyse the data on vulnerability to predation, we used logistic regression to compare the number of tadpoles alive versus dead after 24 h, using ‘suppression treatment’ and ‘day’ (2 versus 10) as fixed effects. The analysis was based on the quasi-binomial distribution. We used a 2-way ANOVA in SPSS (IBM, Armonk, NY) to compare the mass of the tadpoles, with ‘suppression treatment’ and ‘day’ as fixed factors. Tadpole clutch identity was included in both analyses as a random factor.

To analyse the data on trapping rates after alarm treatment, we used logistic regression with the number of tadpoles trapped (versus not trapped) as the dependent variable and with ‘alarm treatment’ and ‘trapping treatment’ as fixed effects. The analysis was based on the quasi-binomial distribution to account for over-dispersion of data.

## Results

### Effects of exposure to the suppression cue

#### Trapping rates

**Fig 1:**
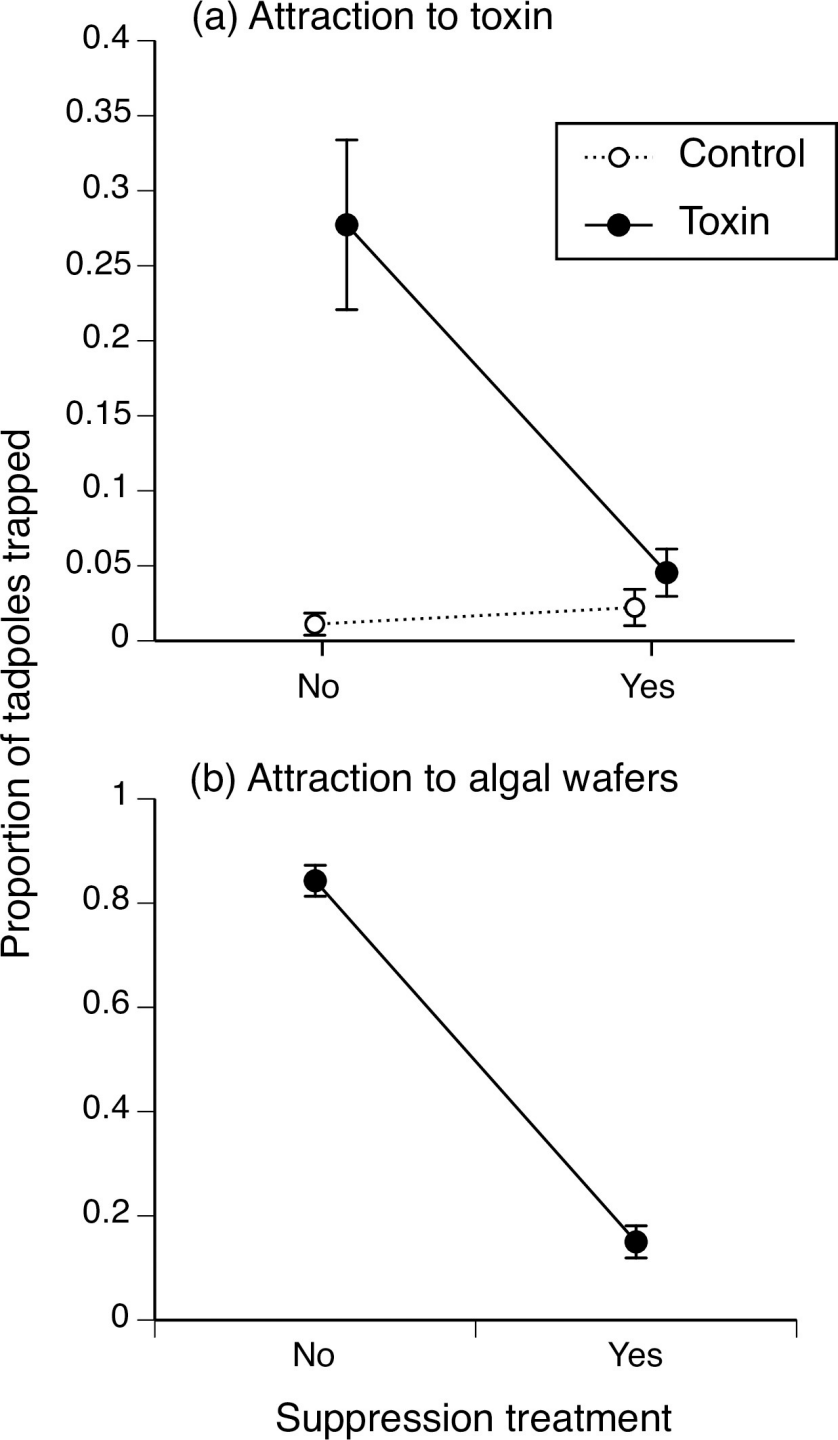
The proportion of non-suppressed and suppressed cane toad tadpoles (*Rhinella marina*) captured in funnel traps after 90 min, when traps were baited with (a) 200 mg of adult toad toxin or unchlorinated bore water (n = 9–11), or (b) 200 mg of algal wafer (n = 7). Suppression-treated tadpoles were exposed to older conspecifics for 48 h during larval development. The graphs show mean values ± SE.

The number of tadpoles trapped was affected by a significant interaction between suppression treatment and trapping treatment (t = 2.21, df = 1, P = 0.03; Fig 1A). When tadpoles were not suppressed, we captured more tadpoles using the toxin bait than the control bait (Fig 1A). However, when tadpoles were suppressed, both treatments caught an equally low number of tadpoles (0–1 of 20; Fig 1A). Tadpoles that were not suppressed responded strongly to the algal food bait, whereas suppressed tadpoles did not (t = 12.15, df = 1, P < 0.001, capturing approximately 16 versus 2 of 20 tadpoles, respectively; Fig 1B).

#### Response to alarm cues

**Fig 2:**
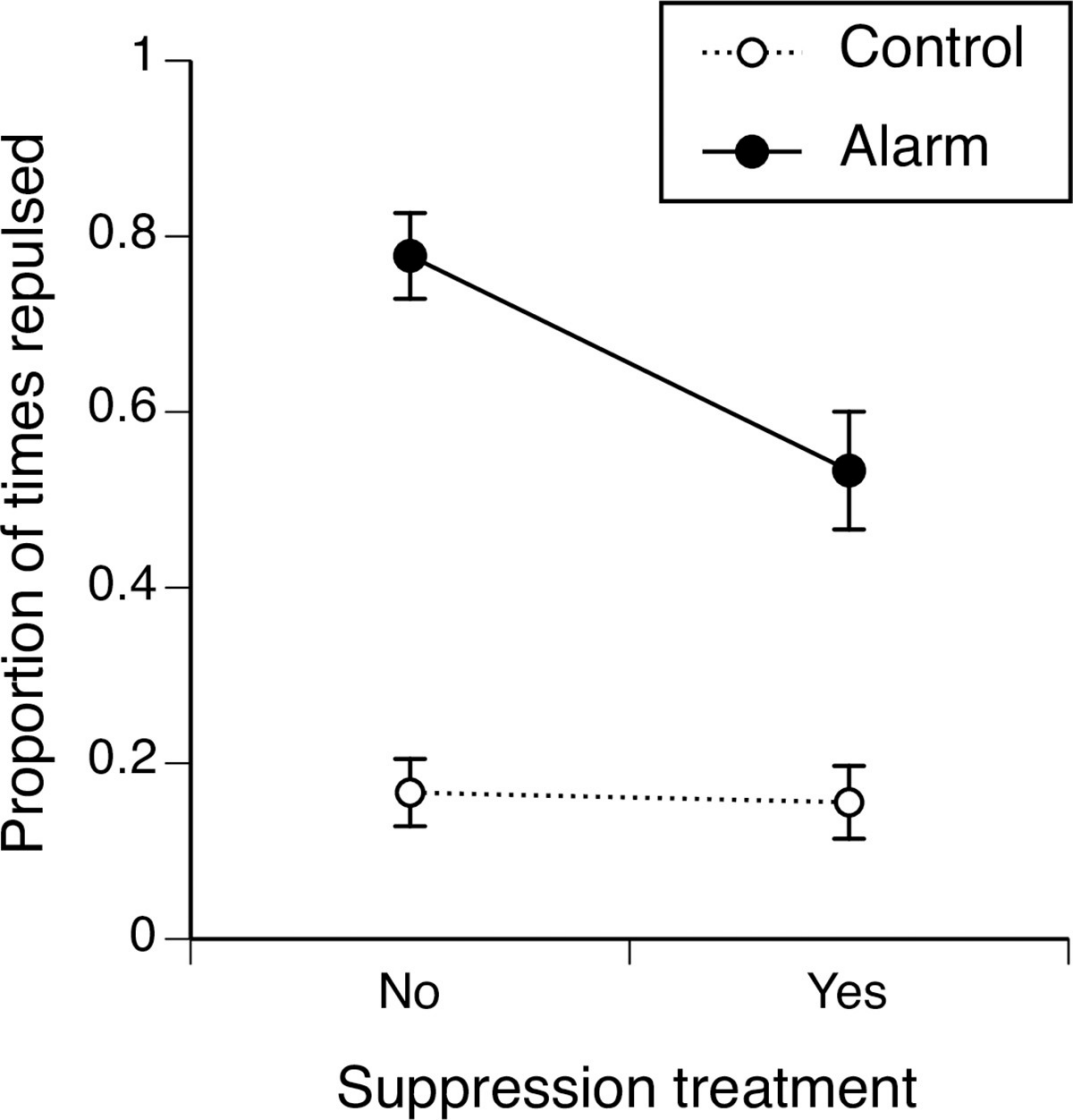
The proportion of times non-suppressed and suppressed cane toad tadpoles (*Rhinella marina*) exhibited a repulsion response when encountering either unchlorinated bore water or conspecific alarm cue (n = 30). Suppression-treated tadpoles were exposed to older conspecifics for 48 h during larval development. The graph shows mean values ± SE.

All tadpoles responded more strongly to the alarm cue than to a control cue (*t* = 6.74, *df* = 1, *P* < 0.001; Fig 2) regardless of suppression treatment (suppression: *t* = 0.18, *df* = 1, *P* = 0.86; interaction: *t* = 1.77, *df* = 1, *P* = 0.08).

#### Vulnerability to predation

**Fig 3:**
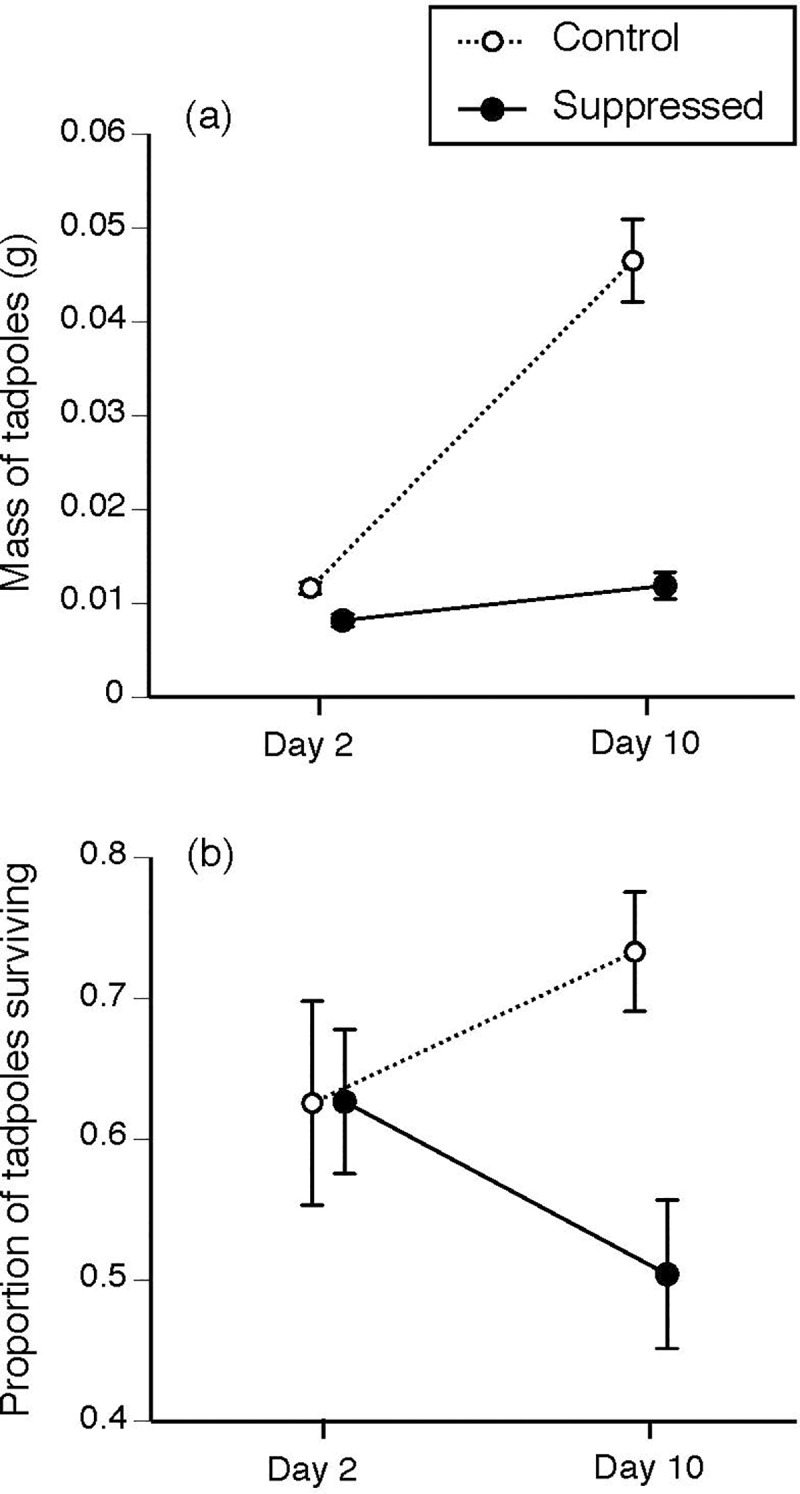
The (a) mass and (b) proportion of cane toad tadpoles (*Rhinella marina*) surviving 24 h of predator exposure, after 2 and 10 days of growth (n = 20 and 24–27 respectively). White circles represent non-suppressed tadpoles, and black circles represent suppressed tadpoles (exposed to older conspecifics for 48 h during larval development). The graphs show mean values ± SE.

The survival and growth of tadpoles was affected by an interaction between the suppression treatment and day (interaction effect, survival: *t* = 1.99, *df* = 1, *P* = 0.049; growth: *F*_1,73_ = 44.85, *P* < 0.0001; Fig 3A and 3B). On Day 2, the proportion of tadpoles surviving in each treatment after 24 h did not differ significantly between treatments (approximately 6–7 of 10), but on Day 10 the suppression treatment had fewer survivors than the control treatment (5 versus 7–8 of 10; Fig 3A). Similarly, on Day 2 there was no significant difference in tadpole mass between treatments, but on Day 10 control tadpoles were heavier than suppressed tadpoles (Fig 3B).

### Effects of exposure to the alarm cue

**Fig 4:**
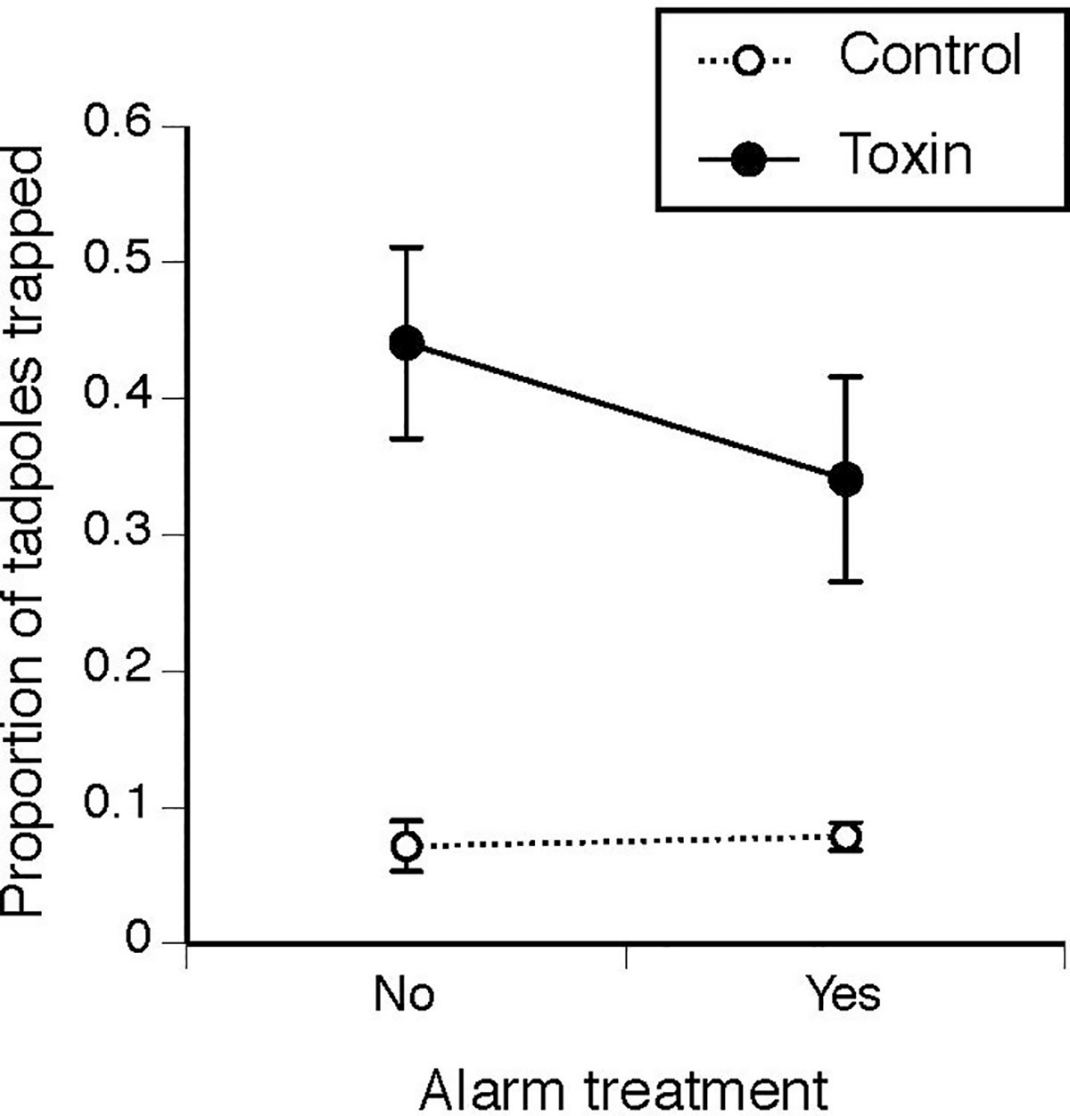
The proportion of alarm-treated and non-alarm-treated cane toad tadpoles (*Rhinella marina*) captured in funnel traps after 90 min, when traps were baited with either 200 mg of adult toad toxin or unchlorinated bore water (n = 7–11). Alarm-treated tadpoles were exposed to freshly crushed conspecifics daily for 7 days before testing. The graph shows mean values ± SE.

Toxin traps caught more tadpoles than did control traps (*t* = 3.62, *df* = 1, *P* = 0.001; approximately 6–8 versus 1 of 20), regardless of alarm treatment (alarm: *t* = 0.125, *df* = 1, *P* = 0.9; interaction: *t* = 0.59, *df* = 1, *P* = 0.56; Fig 4).

## Discussion

Our results support previous reports on the response of cane toad tadpoles to chemical cues; that is, tadpoles were suppressed by the suppression cue (Clarke *et al*. [18]), repulsed by the alarm cue [23] and drawn to the attraction cue (Crossland *et al*. [17]). However, the response to combinations of these cues was varied, and demonstrates the need to consider cumulative effects if multiple cues are to be used as a combined control strategy.

Firstly, exposure to the suppression cue during larval development eliminated the tendency for cane toad tadpoles to approach the attraction cue or food, resulting in low capture rates in baited traps. Previous histological comparisons of suppressed and non-suppressed tadpoles have reported significantly delayed development in suppressed tadpoles, particularly in the posterior kidney, hind limb and central nervous system (Clarke *et al*. [18]). We hypothesise that the physiological systems affected by suppression are used in the detection of attraction cues, and that stunted development in tadpoles results in an inability to detect, identify and /or respond to this information. Although suppressed tadpoles are still able to ingest food (S. McCann, *personal observation* 2016), an inability to detect food sources likely would lead to a decrease in consumption of food, exacerbating their already delayed growth. Although the specific physiological and morphological pathways by which conspecific suppression affects tadpole growth and increases mortality is not yet understood, our results suggest that delayed development of tadpoles (as induced by suppression) impacts their ability to detect and/or respond to chemical cues, reducing the viability of suppressed individuals.

Interestingly, suppressed tadpoles were repulsed by the alarm cue as strongly as were control tadpoles, indicating that suppression does not remove the ability to detect all conspecific cues. However, cane toad tadpoles respond to the alarm cue from a much earlier stage of development than they do to the attraction cue (alarm cue, Gosner stage 21: [30]; attraction cue, Gosner stage 27–28: S. McCann, *unpublished data*). This disparity suggests that the physiological systems used in detecting and responding to alarm cues may already be established by the time the suppression cue begins to affect ontogenetic development.

Tadpoles exposed to the suppression cue during larval development were smaller than control tadpoles at 10 days post-hatching, and consequently, were more vulnerable to an aquatic invertebrate predator. Relative body sizes play a major role in regulating interactions in many predator–prey relationships, influencing density, size distributions and relative survival of the prey [31]. The pattern we observed (increased vulnerability of smaller tadpoles) is commonly seen in tadpole predator–prey studies [31–33], and is attributed to the increased likelihood of larger tadpoles escaping attempted predator strikes (rather than predators not attempting to capture larger prey [33]). An increased likelihood of escape by larger tadpoles may be due to the difficulty of grasping larger individuals, or the increased sprint-speed of larger individuals allowing for quicker escape [33, 34]. Due to our experimental design it is difficult to determine whether suppressed tadpoles are more frequently consumed by predators due to size alone, or if other attributes of suppressed individuals (e.g. delayed development of the nervous system and consequent behaviours) play a role. Regardless, the pheromonal suppression of cane toad tadpoles renders them more vulnerable to aquatic predators, further reducing tadpole survival.

Exposure to the alarm cue did not inhibit a tadpole’s response to the attraction cue, with alarm cue-treated tadpoles captured in toxin-baited traps as frequently as were non-exposed individuals. In contrast, exposure to the alarm cue decreased the feeding response of tadpoles of the American toad *Anaxyrus americanus* [35]. Why did we obtain a different result? Firstly, cane toad tadpoles habituate to the alarm cue after two hours of regular exposure, with a negligible response after four hours of exposure [30]. Potentially, our alarm cue-treated tadpoles had habituated to alarm chemicals by the time trapping was conducted, and thus no longer responded to predation risk when presented with an attraction cue. Alternately, the ‘reward’ of the attraction cues presented may have outweighed the ‘risk’ associated with the alarm cue, particularly as tadpoles were responding to the cue of conspecific eggs as opposed to regular food. The elimination of conspecific eggs (mimicked by the scent of toxin) not only provides tadpoles with food, but also eliminates future competitors [25], creating a stronger signal than food alone. The toad tadpoles may have responded to the stronger cue when presented with alarm and attraction cues in combination, suggesting that a stronger alarm cue or a less concentrated attraction cue may affect the patterns observed.

Our results have important implications if tadpole control strategies are to be appropriately combined and employed. Firstly, the ability of the suppression cue to eliminate a tadpole’s affinity to the attraction cue indicates that combining suppression with baited trapping (arguably a logical “next step” in tadpole control) would have little benefit beyond that achieved by using one of these strategies alone. However, suppression removed the ability of tadpoles to respond to food cues in general, meaning that survival of suppressed tadpoles in the field may be much lower than in previous laboratory studies, due to the suppressed animals’ inability to locate food. Most suppression experiments thus far have been conducted in 1 L containers, and tadpoles have been provided with fresh food daily, requiring little to no ability to seek it out [18, 21, 25]. It would be interesting to determine how survival is affected when suppressed tadpoles are raised in a larger volume of water, where food is more difficult to locate. If suppression reduces a tadpole’s ability to find food, the suppression cue could be more effective in lowering tadpole survival than has been inferred from previous laboratory studies.

Exposure to the suppression cue also rendered tadpoles more vulnerable to predation, due to their significantly reduced size. This result suggests that if suppression were to be applied in the field, survival of the growth-suppressed tadpoles would be further decreased by invertebrate predators. The remarkable success of cane toads in Australia is at least partially attributable to their potent bufotoxins [26], which are fatal if ingested by vulnerable species of frog-consuming predators (including mammals, reptiles, amphibians and invertebrates: see [14]). However, many invertebrate predators are unaffected by bufotoxins [36, 37], and can consume toad tadpoles without deleterious effects (e.g. the water beetle *Cybister godeffroyi* and water bugs *Diplonychus rusticus* and *Lethocerus insulanus* [38]). Cane toad larvae already encounter a high risk of predation in many parts of Australia [38], exacerbated by seasonal fluctuations in depth of water bodies, and consequent high densities of aquatic predators when water levels fall. Choice experiments have demonstrated that some invertebrate predators actively prefer cane toad tadpoles to native tadpoles, most likely due to the toads’ smaller size and slower movement [38]. Thus, suppression as a field-control strategy has the potential to reduce the survival of cane toad tadpoles not only through reductions in growth and survival, but also via increased susceptibility to predation.

The next step in this research area is to take it out of the laboratory and into the field: that is, to determine how the interactions between cane toad chemical cues play out under field conditions. Such a field study could usefully explore how each of the currently proposed tadpole control strategies (suppression, attraction trapping, alarm cue, and combinations of these) function under field levels of predation, environmental variation and density, in order to facilitate informed decisions about future control strategies. More broadly, our results demonstrate the importance of measuring the impact of combinations of pest control approaches on overall effectiveness, before such integrated strategies are applied on a large scale. Carefully designed experimental trials and subsequent field studies are essential if we are to understand how best to allocate resources and maximise the effectiveness of invader control.
